# Trajectories of Mediterranean Diet Adherence and Risk of Hypertension in China: Results from the CHNS Study, 1997–2011

**DOI:** 10.3390/nu10122014

**Published:** 2018-12-19

**Authors:** Min Gao, Fengbin Wang, Ying Shen, Xiaorou Zhu, Xing Zhang, Xinying Sun

**Affiliations:** Department of Social Medicine and Health Education, School of Public Health, Peking University Health Science Center, Beijing 100191, China; vivianhbs@pku.edu.cn (M.G.); fbwang@pku.edu.cn (F.W.); shenying@bjmu.edu.cn (Y.S.); zxr@bjmu.edu.cn (X.Z.); zhangxing@bjmu.edu.cn (X.Z.)

**Keywords:** Mediterranean diet, group-based trajectory modeling, hypertension, blood pressure

## Abstract

Evidence indicates that longitudinal changes in dietary patterns may predict variations in blood pressure (BP) and risk of incident hypertension. We aimed to identify distinct trajectories in the levels of Mediterranean diet adherence (MDA) in China and explore their association with BP levels and hypertension risk using the China Health and Nutrition Survey 1997–2011 data. Three levels of MDA were constructed. The trajectories in these levels were constructed using group-based trajectory modeling. A Cox proportional hazards model was used to measure the association between MDA trajectory groups and the risk of incident hypertension after adjusting for covariates. Finally, 6586 individuals were included. Six distinct MDA trajectory groups were identified: persistently low and gradual decline; rapidly increasing and stabilized; persistently moderate; slightly increasing, steady, and acutely descending; slightly decreasing and acutely elevated; and persistently high. The systolic BP and diastolic BP were significantly lower in trajectory groups with rapidly increasing and stabilized MDA; slightly increasing, steady, and acutely descending MDA; and persistently high MDA. Cox regression analysis showed that the risks of developing hypertension were relatively lower in the group with slightly increasing, steady, and acutely descending MDA (hazard ratio (HR) = 0.17, 95% confidence interval (CI): 0.09–0.32) and the group with rapidly increasing and stabilized MDA (HR = 0.32, 95% CI: 0.23–0.42), but the risk was the highest in the trajectory with persistently moderate MDA (HR = 0.96, 95% CI: 0.84–1.08). In conclusion, MDA in China was categorized into six distinct trajectory groups. BP was relatively lower in trajectory groups with initially high or increasing MDA levels. Greater MDA was significantly associated with a lower risk of developing hypertension.

## 1. Introduction

The Mediterranean diet (MD) is receiving increasing attention due to its association with the prevention of cardiovascular events. The MD is characterized by a high intake of olive oil and nuts, cereals, fruit and vegetables; moderate intake of fish, poultry and wine with meals; and low intake of red and processed meats, dairy products, and sweets [1], and may serve as a healthy dietary pattern to prevent health outcomes including cardiovascular disease and metabolic syndrome in adults [2,3].

Several studies have documented that the MD could increase the likelihood of controlling blood pressure (BP) [4,5]. The MD is rich in components presumed to be beneficial to health, including vegetables, legumes, fruits, nuts, cereals, and fish and features a moderate intake of meat, alcohol and dairy products. The MD is beneficial to health because these fresh foods are rich in fiber, antioxidant polyphenols, and essential micronutrients and macronutrients [6]. In a small clinical study, a high degree of adherence to the MD could reduce BP [7]. Recently, the results of a meta-analysis including clinical studies indicated that adhering to the MD is health-protective for systolic BP (SBP) and diastolic BP (DBP) [2]. Greater reductions in SBP and DBP were observed in the intervention (MD) group compared to those in the control group [8]. More specifically, a meta-analysis including 2650 individuals found that after 2 years of follow-up, individuals on the MD had more favorable changes in SBP (−1.7 mmHg; 95% confidence interval (CI): −3.3 to −0.05) and DBP (−1.5 mmHg; 95% CI: −2.1 to −0.8) [9]. Abnormal BP levels have been identified as a consistent risk factor for cardiovascular events [10]. Many studies have also reported that higher adherence to the MD could reduce the risk of hypertension [11,12].

Accompanied with rapid economic changes, the dietary pattern in China is changing from a high intake of cereals and vegetables and low intake of animal food to the Western pattern with a high intake of animal foods and other high-energy-density foods [13,14]. Hypertension is one of the most serious chronic diseases in China, with approximately 270 million hypertension cases in 2017 [15]. Dietary lifestyle plays an important role in controlling BP and the development of hypertension [9]. Several convincing studies, in the social context of Western developed countries, have investigated the association between diet and the development of hypertension [16,17]. The mechanisms underlying the relationship between MD and cardiovascular disease include improved lipid profiles and reduced BP [18]. 

Therefore, the aims of this study were to investigate (1) the secular trends of dietary patterns in a Chinese population, (2) the association between MD adherence (MDA) trajectories and BP values over time, and (3) the association between different trajectory groups and the risk of developing hypertension. 

## 2. Materials and Methods 

### 2.1. Study Population

The China Health and Nutrition Survey (CHNS), an ongoing large-scale and household-based survey, was started in 1989, with follow-up every 2–4 years. A multistage random-cluster sampling design was utilized to select samples from nine provinces in mainland China that vary in demography, geography, economic development, and public resources. The CHNS data cover 228 communities in nine diverse provinces throughout China (North: Heilongjiang and Liaoning; Central: Shandong, Henan and Jiangsu; and South: Hunan, Hubei, Guangxi, and Guizhou). More details are described on the survey website (http://www.cpc.unc.edu/projects/china/about/proj_desc/survey). The present study included 16,030 participants aged 18 years and over in 1997. Participants who were diagnosed with hypertension at the baseline (*n* = 1767), who were lost to follow-up (*n* = 5074), and without complete data regarding blood pressure, diet, sex, age, exercise, or smoking (*n* = 2603) were excluded. Through 2011, a total of 6586 individuals were retained (see in Figure S1). The CHNS was approved by the ethics committee of the National Institute of Nutrition and Food Safety of the Chinese Center for Disease Control and Prevention and the Institutional Review Board of the University of North Carolina at Chapel Hill.

### 2.2. Measurements

#### 2.2.1. Dietary Assessment

The dietary intake of each participant was assessed using 24-h recalls for three consecutive days (2 weekdays and 1 weekend day), supplemented with a household food inventory weighing method to estimate the consumption of cooking oils and condiments. Researchers at the CHNS recorded the types and amounts of food consumed at each meal during the previous day. Detailed information on data collection has been reported previously [19,20]. We used the MD score scale proposed by Trichopoulou [11]. This scale includes nine components: vegetables, legumes, fruits, nuts, cereals, fish, meat and meat products, dairy products, and alcohol. Values of 0 or 1 were assigned to each of the components, using the sex-specific median values for the participants as cutoffs for all components except for meat, alcohol, and dairy. For the six components presumed to be beneficial (vegetables, legumes, fruits, nuts, cereals, and fish), participants whose intake was at or above the sex-specific medians were assigned a value of 1, while those with intake below these medians were assigned a value of 0. For meat, a value of 0 was assigned to participants whose intake was below the median, while those whose intake was below the sex-specific median were assigned a value of 1. For alcohol intake, a value of 1 was assigned to men who consumed 10–50 g/day and women who consumed 5–25 g/day. For dairy products, a value of 1 was assigned to those whose daily intake was between 5 g and 25 g. The scores of the nine categories were summed. The total range in MD score was from 0 to 9. The MD score was calculated for all individuals upon recruitment. Adherence to the MD was categorized into three levels: low (score 0–2), moderate (score 3–6) or high (score 7–9) [21], using the low level as the reference level.

#### 2.2.2. Blood Pressure and Hypertension

Each participant’s SBP and DBP were measured on the right arm with the lower edge of the cuff placed ~25 mm above the elbow after a 10-min seated rest using mercury sphygmomanometers. Blood pressure was measured three times at 30-s intervals. The mean of the three measurements was used. During an interview, participants were asked whether they had been diagnosed with hypertension. In this study, participants were considered to have hypertension if they had been diagnosed with hypertension by a physician, or had an average SBP of at least 140 mmHg or a DBP of at least 90 mmHg. 

#### 2.2.3. Other Variables

Age group (18–29, 30–39, 40–49, 50–59, 60–69, and 70+ years) and sex (male or female) were included. Lifestyle factors were also assessed. In accordance with the Chinese Adult Physical Activity Guide, participants who achieved 10 MET/h per week were assigned a value of 1. Otherwise, those who did not achieve the exercise goal were assigned a value of 0. Smoking status was divided into current (coded as 1) and noncurrent (coded as 0) according to the interview responses of participants.

### 2.3. Statistical Analysis

To obtain more information on the relationships between changes in levels of MDA and BP values, a group-based trajectory model (GBTM) was used to identify similar developmental trajectories in the levels of MDA. The survey wave was used as a timescale for the trajectories. We modeled the MDA trajectories among participants who were recruited in all the waves. First, a base model without covariates was constructed to determine the number of groups and the order of the polynomial functions of the survey wave. The best-fitting model was considered the trajectory group with the highest probability, which was based on goodness-of-fit statistics using the Bayesian information criterion (BIC). The BIC results showed that the model with six groups with up to quadratic order terms fitted the best (the BIC results are presented in Table S1). Second, each participant was assigned to the corresponding trajectory group based on the maximum likelihood estimation to estimate the probability of producing variance in the MDA of each group. These results enabled us to analyze the relationship between MDA trajectories and abnormal BP. Third, the best-fitted model (six groups, quadratic functions) was further adjusted for other covariates, including sex, age, smoking status, and exercise, and the BP estimations were compared among different trajectory groups. To ensure the robustness of the MDA grouping, we further conducted sensitivity analysis with numbers of trajectory groups having one less and one more than the best-fitted model with the lowest BIC. Fourth, a random effects model was adopted to estimate differences in BP changes with respect to that reference group with adjustment for all covariates. 

Finally, a Cox proportional hazards model was further adopted to measure the association between six MDA trajectory groups and the risk of incident hypertension, with adjustment for age, sex, smoking, and exercise. To account for the trend of included variables over time, all control variables except for sex were considered to be time-varying. Considering that 2603 individuals were excluded, we conducted another sensitivity analysis to estimate the influence of the excluded samples on the research results. An inverse probability weight was adopted to address selection bias, which strengthened the robustness of this study.

## 3. Results

### 3.1. Trajectories of MDA

**Figure 1:**
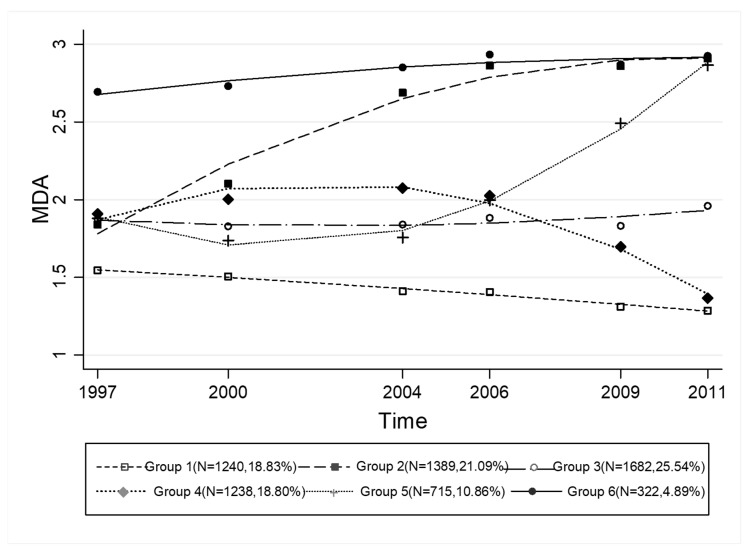
MDA trajectory groups showing differences across waves (6 groups).

Six distinct MDA trajectories were identified during the 15-year period (Figure 1): Group 1 (persistently low and gradual decline, *n* = 1240, 18.83%) referred to those who had lower-than-average MDA profiles during all the waves; Group 2 (rapidly increasing and stabilized, *n* = 1389, 21.09%) represented individuals with a moderate MDA at initial presentation but which increased acutely until 2009 and was stabilized thereafter; Group 3 (persistently moderate, *n* = 1682, 25.54%) had a moderate and steady MDA throughout the entire survey; Group 4 (slight increase, steady, and acutely descending, *n* = 1238, 18.80%) represented individuals with a moderate MDA at the start which increased to 2000, was steady before 2004, and then decreased rapidly. Group 5 (slight decline and acute elevation, *n* = 715, 10.86%) started with a moderate MDA, experienced a slight decrease until 2000, a gradual increase from 2000 to 2004, and then a rapid increase. Group 6 (persistently high, *n* = 322, 4.89%) was characterized by a high MDA at the initial presentation and gradual increase.

Sensitivity analyses to estimate the relationship between five or seven trajectory groups showed similar trends. Detailed information is shown in Figures S2 and S3.

### 3.2. Participants’ Characteristics by Trajectory Groups

The participants in the six MDA trajectory groups had distinct clinical profiles as shown in Table 1. Significant differences in sex, age, smoking, and exercise among the six groups were observed. The proportion of men was largest in Group 6 (58.75%). Participants in Group 6 were also the youngest (mean age, 20.50 years), while participants in Groups 1 and 3 were relatively older (mean age, 38.13 and 38.42 years, respectively). In Group 2, participants were relatively younger (mean age: 25.00 ± 20.86 years). In Group 3, 59.93% of participants had accomplished the Chinese-recommended amount of exercise (10 MET/h per person per week).

### 3.3. Trajectories of MDA and BP Change

To further estimate the BP distributions among the six trajectories, a random effects model was adopted to describe the BP change among the six groups (see Table 2). After adjusting for confounding factors (sex, age, smoking, and exercise), the MDA trajectory in Group 2 was significantly associated with a larger reduction in SBP and DBP values (Coef. = −13.40 and 7.57, respectively, *p* < 0.001). The MDAs in Group 6 (Coef. = −8.44, *p* < 0.001) and Group 5 (Coef. = −7.75, *p* < 0.001) were inversely associated with SBP values. In addition, significantly negative associations between MDA and DBP values were observed in Group 5 (Coef. = −4.50, *p* < 0.001) and Group 6 (Coef. = −3.64, *p* < 0.001). While the SBP and DBP were negatively associated with MDA in Groups 3 and 4, the difference was not significantly significant (*p* > 0.5). Individuals in Groups 1, 3, and 4 shared more similarities, including relatively older ages, higher prevalence of current smoking, and a higher proportion having achieved the recommended amount of exercise.

Sensitivity analyses of the estimated relationship between five- or seven-trajectory groups for MDA and BP values showed similar patterns. The distributions of these trajectory groups and their corresponding BP values are shown in Tables S2 and S3. 

### 3.4. MDA and the Risk of Hypertension

The Cox proportional hazards models showed that a variation in MDA was significantly associated with a lower risk of incident hypertension (Table 3). During the follow-up period, 1109 participants were identified as having hypertension. Treating Group 1 as the reference, the age- and sex-adjusted hazard ratios (HRs) were 0.29 for Group 2 and 0.96 for Group 3. After adjusting for age, sex, smoking, and exercise, the effect sizes of MDA were slightly increased in Groups 2, 5, and 6. The multivariate-adjusted model suggested that the risk of developing hypertension was lowest in Groups 6 (HR = 0.17, 95% CI: 0.09–0.32) and 2 (HR = 0.32, 95% CI: 0.23–0.42), followed by Group 5 (HR = 0.75, 95% CI: 0.63–0.91). Group 3 showed the highest risk of developing hypertension (HR = 0.96, 95% CI: 0.84–1.08). 

Sensitivity analysis was also conducted to estimate the influence of the excluded samples (*n* = 2603) on the results (Table S4). 

## 4. Discussion

To the best of our knowledge, this was the first study to report the Chinese dietary pattern and six distinct trajectories of MDA in a large cohort of the Chinese population followed for 15 years. BP was relatively lower in groups with initially high or increasingly higher MDA levels. Moreover, a life course approach indicated that these levels were significantly associated with a lower risk of developing hypertension after adjusting for other covariates.

The distributions of Chinese MDA were classified into six trajectory groups. Different MDA trajectories increased linearly with time. Figure 1 shows that people with consistently moderate adherence to the MD accounted for a large proportion of the included participants (*n* = 1682). Increasing trends in MDA were observed in 2426 individuals compared to 2478 individuals with a declining MDA. Over the past 20 years, the dietary patterns in China have transitioned from a traditional diet including high intakes of rice, vegetables, poultry, pork, and fish to a modern diet consisting of high intakes of wheat, processed meat, and fast foods [22,23]. Table 1 shows that people of younger age were less likely to smoke, consistent with evidence from the 2015 China Adult Tobacco Survey, whereas people of higher age may have more leisure time and are more likely to achieve the recommended 10 MET/h per person per week. 

Many studies have reported the association between MD and a lower BP [8,24,25]. A high MD score was significantly associated with lower SBP variation [26]. A large-scale Spanish cohort study reported that MDA was inversely associated with SBP and DBP. Among participants without hypertension, adopting the MD could reduce SBP and DBP by 3.5 and 2.1 mmHg, respectively [21]. A recent meta-analysis that pooled six trials reported that encouraging people to adopt the MD pattern for at least 1 year could reduce SBP and DBP levels in individuals with normal BP or mild hypertension [27]. The PREDIMED study was a nutritional intervention trial with a randomized parallel design, the detailed information of which has been reported elsewhere [28]. Studies have reported decreased SBP and DBP in MD groups [6,8]. A 1-year randomized clinical trial showed that 1 year after adopting a supplemented MD, the MD with olive oil group had mean SBP and DBP reductions of 2.3 and 1.2 mmHg, respectively; in the MD with nuts group, the changes were 2.6 and 1.2 mmHg, respectively [29]. Another large randomized controlled trial indicated that the traditional MD could exert beneficial effects on BP control, but in groups promoting the MD with extra virgin olive oil or nuts, the DBP values were relatively lower [6]. A cross-sectional study of sex differences in the adherence to the French Nutrition and Health Program policies reported that adherence to the nutritional recommendations was inversely associated with BP levels in women, but no relationship between dietary scores and BP level in men was observed [30].

In the present study, the BP levels were lower in trajectory groups with increasingly higher or initially higher MDA scores. A large-scale prospective investigation in Greece found that a greater adherence to the traditional MD was associated with a significant reduction in total mortality [11]. The Dietary Approaches to Stop Hypertension (DASH) trial was another important multicenter, randomized feeding study that tested the combined effects of dietary patterns rather than single nutrients on BP. The DASH-style diet, which shared many constituents with those of Mediterranean-type diets, was a combination diet based on high intakes of fruit and vegetables and low intakes of fat and sodium. Some studies based on the DASH trial found a beneficial effect of the DASH-style diet on BP reduction [31,32]. In our study, trajectory groups with initially or increasingly higher MDA levels could be an independent predictor of uncontrolled BP and may help to identify patients at high risk of future hypertension events. 

Relationships between MD adherence and a reduced incidence of other chronic diseases have recently been reported [33]. The results of Cox regression in the present study showed that a higher MDA could confer a reduced risk of hypertension, consistent with other findings [11,12]. A reduction in BP served as an underlying mechanism between the MDA and the incidence of cardiovascular disease [8,34]. In our study, groups with initially high or increasingly higher MDA levels tended to have a reduced BP; in addition, these groups tended to have a lower risk of developing hypertension. However, a Spanish cohort study did not find evidence of a negative association between MDA and incident hypertension. The difference in food habits between groups was not clear and may produce between-subject homogeneity in exposure and further bias the results [21]. 

To the best of our knowledge, this was the first study to classify MDA in a Chinese population into six trajectory groups and explore the relevance between MDA trajectories and changes in BP. Many previous studies used baseline dietary habits as the relevant exposure, ignoring the dynamic changes in diet during follow-up. This lack may conceal the effect of changing dietary patterns. The present study adopted a group-based trajectory model to classify the MDA patterns into groups sharing common dietary characteristics. Second, a sensitivity analysis was conducted to classify MDA trajectories into five or seven groups to compare the differences among six groups. In addition, we compared the differences in BP changes in the five or seven group classifications of trajectories, which could increase the robustness of our study.

One limitation of our study was that the accurate time of developing hypertension could not be collected from the dataset because participants reported only whether they had hypertension during their interview. We defined the time of developing hypertension as the time when participants reported having had hypertension. Therefore, the results may be biased because of inaccurately reported time. Another limitation was that the reverse effect of BP on diet cannot be excluded. In other words, it is possible that people may begin to change their diet after noticing an increasing BP. Finally, other health determinants were not included in this study, which may bias our results.

These findings have implications for intervention and prevention of high BP among individuals who were not diagnosed with hypertension. From a public health perspective, there is an urgent need for approaches to advocating the adoption of Mediterranean-style diets to tackle uncontrolled BP and hypertension issues. For example, Mediterranean-style diets, one of the main approaches of the DASH diet program, has a protective effect against cardiovascular events [12]. In China, health promotion departments should encourage the early adoption of Mediterranean-style diets to further prevent the development of hypertension.

## 5. Conclusions

The MDA levels in the Chinese population during a 15-year period could be classified into six trajectories. In trajectories with initially high or increasing MDA levels, the BP and risk of developing hypertension were significantly lower. Promoting the adoption of a Mediterranean-style diet early could be an effective way to prevent incident hypertension. 

## Figures and Tables

**Table 1:** Participants’ characteristics by trajectory groups at the baseline.

Group	Group 1	Group 2	Group 3	Group 4	Group 5	Group 6	chi^2^/F	*p* Value ^b^
*n*	1240 (18.83%)	1389 (21.09%)	1682 (25.54%)	1238 (18.80%)	715 (10.86%)	322 (4.89%)		
Gender (%)							30.8	<0.001
Female	48.79%	43.52%	52.51%	45.67%	48.55%	41.25%		
Male	51.21%	56.48%	47.49%	54.33%	51.45%	58.75%		
Age ^a^	38.13 ± 13.92	25.00 ± 20.86	38.42 ± 17.27	34.54 ± 15.82	26.21 ± 10.20	20.50 ± 8.45	460.97	<0.001
Smoking status (%)							227.14	<0.001
Noncurrent smokers	71.31%	81.97%	73.40%	71.82%	90.77%	84.69%		
Current smokers	28.69%	18.03%	26.60%	28.18%	9.23%	15.31%		
Exercise							665.33	<0.001
Had not achieved 10 MET/h per person per week	51.22%	68.03%	40.07%	46.52%	80.38%	74.37%		
Had achieved 10 MET/h per person per week	48.78%	31.97%	59.93%	53.48%	19.62%	25.63%		

**Note:**^a^ Values are expressed as mean ± standard deviation. ^b^ Significant differences were found in gender, age, smoking status, and exercise (*p* < 0.001); X^2^ tests for dichotomous variables and one-way ANOVA test for continuous variables.

**Table 2:** SBP and DBP values according to 6 trajectories of MDA.

	SBP				DBP			
	Coef.	*p* Value	95% CI		Coef.	*p* Value	95% CI	
Low Adherence (reference group)	0.00				0.00			
Group 2	−13.40	<0.001	−14.51	−12.29	−7.57	<0.001	−8.26	−6.89
Group 3	−0.12	0.803	−1.04	0.80	−0.03	0.908	−0.59	0.53
Group 4	−0.65	0.328	−1.97	0.66	0.11	0.782	−0.68	0.91
Group 5	−7.75	<0.001	−8.90	−6.59	−4.50	<0.001	−5.20	−3.79
Group 6	−8.44	<0.001	−11.15	−5.72	−3.64	<0.001	−5.33	−1.95

Coef., regression coefficient; CI, confidence interval；SBP, systolic blood pressure; DBP, diastolic blood pressure; MDA, Mediterranean diet adherence. All regression models have been adjusted for gender, age, smoking, and exercise.

**Table 3:** Hazard ratios for hypertension according to MDA and changes in MDA.

	Age- and Gender-Adjusted ^a^			Multivariate-Adjusted ^b^		
	HR	95% CI		HR	95% CI	
Group 1 (reference group)	1.00			1.00		
Group 2	0.29	0.22	0.39	0.32	0.23	0.42
Group 3	0.96	0.85	1.09	0.96	0.84	1.08
Group 4	0.92	0.77	1.10	0.92	0.77	1.10
Group 5	0.73	0.61	0.88	0.75	0.63	0.91
Group 6	0.15	0.08	0.29	0.17	0.09	0.32

**Note:** CI, confidence interval; HR, hazard ratio; MDA, Mediterranean diet adherence ^a^ Adjusted for gender and age; ^b^ Adjusted for gender, age, smoking, and exercise.

## Supplementary Materials

The following are available online at http://www.mdpi.com/2072-6643/10/12/2014/s1, Figure S1: Flow chart of the China Health and Nutrition Survey analytic sample. Table S1:Estimation process for the trajectory groups of MDA. Figure S2: MDA showing differences across waves (5 groups). Figure S3: MDA showing differences across waves (7 groups).Table S2: SBP and DBP values according to 5 trajectories of MDA. Table S3: SBP and DBP values according to 7 trajectories of MDA. Table S4: Hazard Ratios for Hypertension According to MDA and changes in MDA.

## References

[1] Willett W.C., Sacks F., Trichopoulou A., Drescher G., Ferroluzzi A., Helsing E., Trichopoulos D. (1995). Mediterranean diet pyramid: A cultural model for healthy eating. Am. J. Clin. Nutr..

[2] Kastorini C.M., Milionis H.J., Esposito K., Giugliano D., Goudevenos J.A., Panagiotakos D.B. (2011). The effect of Mediterranean diet on metabolic syndrome and its components: A meta-analysis of 50 studies and 534,906 individuals. J. Am. Coll. Cardiol..

[3] Paletas K., Athanasiadou E., Sarigianni M., Paschos P., Kalogirou A., Hassapidou M., Tsapas A. (2010). The protective role of the Mediterranean diet on the prevalence of metabolic syndrome in a population of Greek obese subjects. J. Am. Coll. Nutr..

[4] Panagiotakos D.B., Pitsavos C.H., Chrysohoou C., Skoumas J., Papadimitriou L., Stefanadis C., Toutouzas P.K. (2003). Status and management of hypertension in Greece: Role of the adoption of a Mediterranean diet the Attica study. J. Hypertens..

[5] Psaltopoulou T., Naska A., Orfanos P., Trichopoulos D., Mountokalakis T., Trichopoulou A. (2004). Olive oil, the Mediterranean diet, and arterial blood pressure: The Greek European Prospective Investigation into Cancer and Nutrition (EPIC) study. Am. J. Clin. Nutr..

[6] Toledo E., Hu F.B., Estruch R., Buil-Cosiales P., Corella D., Salas-Salvadó J., Covas M.I., Arós F., Gómez-Gracia E., Fiol M. (2013). Effect of the Mediterranean diet on blood pressure in the PREDIMED trial: Results from a randomized controlled trial. BMC Med..

[7] Perona J.S., Cañizares J., Montero E., Sánchezdomínguez J.M., Catalá A., Ruizgutiérrez V. (2004). Virgin olive oil reduces blood pressure in hypertensive elderly subjects. Clin. Nutr..

[8] Estruch R., Martinezgonzalez M.A., Corella D., Salassalvado J., Ruizgutierrez V., Covas M.I., Fiol M., Gomezgracia E., Lopezsabater M.C., Vinyoles E. (2006). Effects of a Mediterranean-Style Diet on Cardiovascular Risk Factors: A. Randomized Trial. Ann. Int. Med..

[9] Nordmann A.J., Suterzimmermann K., Bucher H.C., Shai I., Tuttle K.R., Estruch R., Briel M. (2011). Meta-Analysis Comparing Mediterranean to Low-Fat Diets for Modification of Cardiovascular Risk Factors. Am. J. Med..

[10] Chobanian A.V., Bakris G.L., Black H.R., Cushman W.C., Green L.A., Izzo J.L., Jones D.W., Materson B.J., Oparil S., Wright J.T. (2003). The seventh report of the joint national committee on prevention, detection, evaluation, and treatment of high blood pressure: The JNC 7 report. JAMA.

[11] Trichopoulou A., Costacou T., Bamia C., Trichopoulos D. (2003). Adherence to a Mediterranean diet and survival in a Greek population. New Engl. J. Med..

[12] Sofi F., Abbate R., Gensini G.F., Casini A. (2010). Accruing evidence on benefits of adherence to the Mediterranean diet on health: An updated systematic review and meta-analysis. Am. J. Clin. Nutr..

[13] Zhai F., Wang H., Du S., He Y., Wang Z., Ge K., Popkin B.M. (2010). Prospective study on nutrition transition in China. Nutr. Rev..

[14] Xu X., Byles J.E., Shi Z., Hall J.J. (2015). Evaluation of older Chinese people's macronutrient intake status: Results from the China Health and Nutrition Survey. Br. J. Nutr..

[15] Ma L., Wu Y., Wang W., Chen W. (2018). Interpretation of the report on cardiovascular diseases in China (2017). Chin. J. Cardiovasc. Med..

[16] Appel L.J., Brands M.W., Daniels S.R., Karanja N., Elmer P.J., Sacks F.M. (2006). Dietary approaches to prevent and treat hypertension: A scientific statement from the American Heart Association. Hypertension.

[17] Oh J., Hong N., Kang S.M. (2010). Dietary therapy in hypertension. New Engl. J. Med..

[18] Appel L.J., Miller E.R., Seidler A.J., Whelton P.K. (1993). Does Supplementation of Diet With 'Fish Oil' Reduce Blood Pressure? A Meta-analysis of Controlled Clinical Trials. JAMA Int. Med..

[19] Xu X., Hall J., Byles J., Shi Z. (2015). Dietary Pattern Is Associated with Obesity in Older People in China: Data from China Health and Nutrition Survey (CHNS). Nutrients.

[20] Wang Z., Gordon-Larsen P., Siega-Riz A.M., Cai J., Wang H., Adair L.S., Popkin B.M. (2016). Sociodemographic disparity in the diet quality transition among Chinese adults from 1991 to 2011. Eur. J. Clin. Nutr..

[21] Núñezcórdoba J.M., Valenciaserrano F., Toledo E., Alonso A., Martínezgonzález M.A. (2009). The Mediterranean diet and incidence of hypertension: The Seguimiento Universidad de Navarra (SUN) Study. Am. J. Epidemiol..

[22] Batis C., Sotres-Alvarez D., Gordon-Larsen P., Mendez M.A., Adair L., Popkin B. (2014). Longitudinal analysis of dietary patterns in Chinese adults from 1991 to 2009. Br. J. Nutr..

[23] Melaku Y.A., Gill T.K., Appleton S.L., Taylor A.W., Adams R., Shi Z. (2017). Prospective Associations of Dietary and Nutrient Patterns with Fracture Risk: A 20-Year Follow-Up Study. Nutrients.

[24] Fung T.T., Mccullough M.L., Newby P.K., Manson J.E., Meigs J.B., Rifai N., Willett W.C., Hu F.B. (2005). Diet-quality scores and plasma concentrations of markers of inflammation and endothelial dysfunction. Am. J. Clin. Nutr..

[25] Thomazella M.C.D., Góes M.F.S., Andrade C.R., Debbas V., Barbeiro D.F., Correia R.L., Marie S.K.N., Cardounel A.J., Daluz P.L., Laurindo F.R.M. (2011). Effects of High Adherence to Mediterranean or Low-Fat Diets in Medicated Secondary Prevention Patients. Am. J. Cardiol..

[26] Lau K.K., Wong Y.K., Chan Y.H., Li O.Y., Lee P.Y., Yuen G.G., Wong Y.K., Tong S., Wong D., Chan K.H. (2015). Mediterranean-style diet is associated with reduced blood pressure variability and subsequent stroke risk in patients with coronary artery disease. Am. J. Hypertens..

[27] Nissensohn M., Románviñas B., Sánchezvillegas A., Piscopo S., Serramajem L. (2016). The Effect of the Mediterranean Diet on Hypertension: A Systematic Review and Meta-Analysis. J. Nutr. Educ. Behav..

[28] Estruch R., Ros E., Salas-Salvadó J., Covas M.-I., Corella D., Arós F., Gómez-Gracia E., Ruiz-Gutiérrez V., Fiol M., Lapetra J. (2013). Primary prevention of cardiovascular disease with a Mediterranean diet. New Engl. J. Med..

[29] Doménech M., Roman P., Lapetra J., García de la Corte F.J., Sala-Vila A., De la Torre R., Corella D., Salas-Salvadó J., Ruiz-Gutiérrez V., Lamuela-Raventós R.-M. (2014). Mediterranean diet reduces 24-hour ambulatory blood pressure, blood glucose, and lipids: One-year randomized, clinical trial. Hypertension.

[30] Lelong H., Blacher J., Menai M., Galan P., Fezeu L., Hercberg S., Kesse-Guyot E. (2016). Association between blood pressure and adherence to French dietary guidelines. Am. J. Hypertens..

[31] Appel L.J., Moore T.J., Obarzanek E., Vollmer W.M., Svetkey L.P., Sacks F.M., Bray G.A., Vogt T.M., Cutler J.A., Windhauser M.M. (1997). A clinical trial of the effects of dietary patterns on blood pressure. New Engl. J. Med..

[32] Harrington J.M., Fitzgerald A.P., Kearney P.M., McCarthy V.J., Madden J., Browne G., Dolan E., Perry I.J. (2013). DASH diet score and distribution of blood pressure in middle-aged men and women. Am. J. Hypertens..

[33] Martínez-González M.Á., De la Fuente-Arrillaga C., Nunez-Cordoba J., Basterra-Gortari F., Beunza J.J., Vazquez Z., Benito S., Tortosa A., Bes-Rastrollo M. (2008). Adherence to Mediterranean diet and risk of developing diabetes: Prospective cohort study. BMJ.

[34] León-Munoz L.M., Guallar-Castillón P., Graciani A., López-García E., Mesas A.E., Aguilera M.T., Banegas J.R., Rodríguez-Artalejo F. (2012). Adherence to the Mediterranean Diet Pattern Has Declined in Spanish Adults–3. J. Nutr..

