# Supplementary material for: Trajectories of Mediterranean Diet Adherence and Risk of Hypertension in China: Results from the CHNS Study, 1997–2011
Source: Nutrients. 2018 Dec 19;10(12):2014. doi: 10.3390/nu10122014 (PMC6315578; doi:10.3390/nu10122014)
Supplement: Supplementary file 1 [file nutrients-10-02014-s001.pdf]

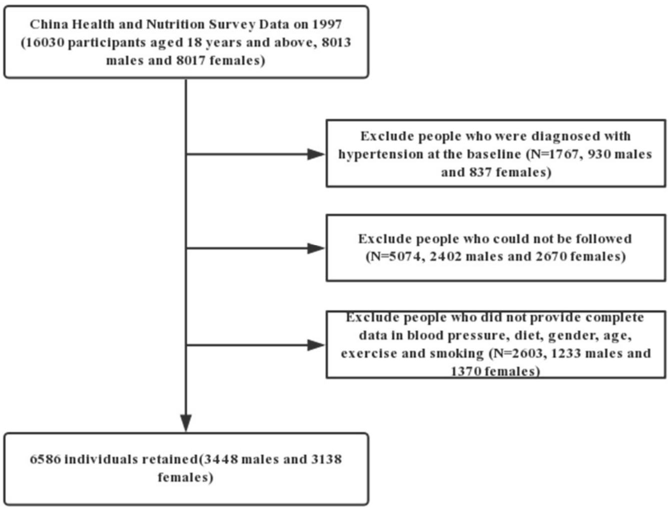

Figure S1. Flow chart of the China Health and Nutrition Survey analytic sample.

Table S1. Estimation process for the trajectory groups of MDA.

Number of Groups	Polynomial	BIC	Proportion of groups (%)						
			Group 1	Group 2	Group 3	Group 4	Group 5	Group 6	Group 7
1	1	−42962.52	100.00						
2	1 1	−38389.28	63.64	36.36					
3	1 1 1	−38081.93	46.00	21.85	32.15				
4	1 1 1 1	−37879.57	45.53	19.12	16.99	18.36			
5	1 1 1 1 1	−37844.81	14.46	22.42	29.82	9.84	23.47		
6	1 1 1 1 1 1	−37809.32	13.43	4.34	34.70	17.75	23.17	6.61	
7	1 1 1 1 1 1 1	−37822.46	13.20	4.28	13.51	17.73	23.16	6.59	21.53
6	2 2 2 2 2 2	−37420.63	19.08	20.17	20.41	22.96	11.18	6.20	
6	3 3 3 3 3 3	−37255.63	16.35	16.17	2.59	19.04	37.06	8.79	
6	4 4 4 4 4 4	−37201.95	18.83	21.09	25.54	18.80	10.86	4.89	

BIC, Bayesian information criterion; MDA, Mediterranean diet adherence.

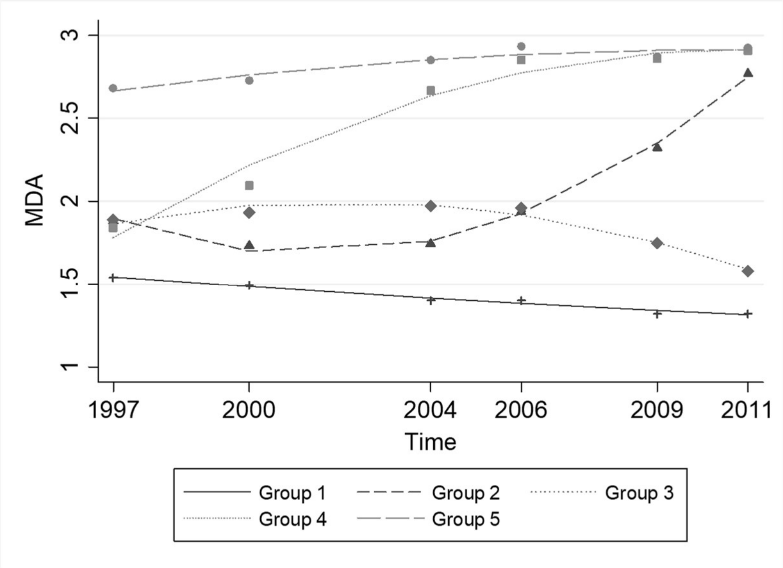

Figure S2. MDA showing differences across waves (5 groups).

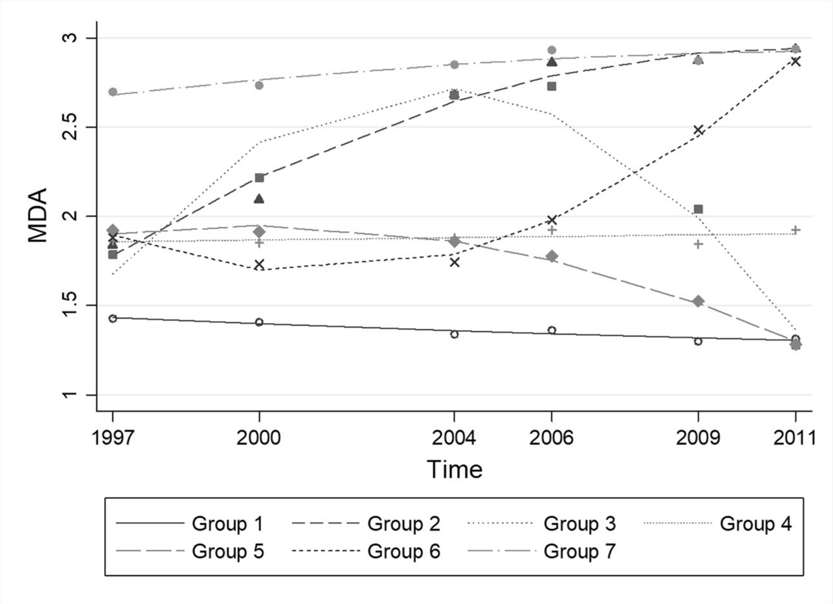

Figure S3. MDA showing differences across waves (7 groups).

Table S2. SBP and DBP values according to 5 trajectories of MDA.

	SBP				DBP			
	**Coef.**	**P**	**95% CI**		**Coef.**	**P**	**95% CI**	
Low Adherence (reference group)	0.00				0.00			
Group 2	-7.49	<0.001	-8.54	-6.45	-4.56	<0.001	-5.20	-3.92
Group 3	-0.26	0.514	-1.05	0.52	-0.28	0.244	-0.76	0.19
Group 4	-13.40	<0.001	-14.40	-12.39	-7.74	<0.001	-8.36	-7.12
Group 5	-8.44	<0.001	-11.11	-5.76	-3.82	<0.001	-5.48	-2.15

Coef., coefficient; CI, confidence interval; SBP, systolic blood pressure; DBP, diastolic blood pressure; MDA, Mediterranean diet adherence.

Table S3. SBP and DBP values according to 7 trajectories of MDA.

	SBP				DBP			
	Coef.	P	95% CI		Coef.	P	95% CI	
Low Adherence (reference group)	0.00				0.00			
Group 2	−14.01	<0.001	−15.08	−12.95	−8.15	<0.001	−8.80	−7.49
Group 3	−6.90	<0.001	−8.79	−5.00	−3.33	<0.001	−4.50	−2.17
Group 4	−0.46	0.317	−1.37	0.44	−0.77	0.006	−1.32	−0.21
Group 5	−0.87	0.080	−1.84	0.10	−0.20	0.517	−0.79	0.39
Group 6	−8.57	<0.001	−9.73	−7.40	−5.16	<0.001	−5.88	−4.45
Group 7	−8.18	<0.001	−10.85	−5.51	−3.84	<0.001	−5.50	−2.18

Coef., coefficient; CI, confidence interval; SBP, systolic blood pressure; DBP, diastolic blood pressure; MDA, Mediterranean diet adherence.

Table S4. Hazard Ratios for Hypertension According to MDA and changes in MDA.

	Age-and Gender-Adjusted [a]			Multivariate-Adjusted [b]		
	HR	95% CI		HR	95% CI	
Low Adherence (reference group)	1.00			1.00		
Group 2	0.35	0.27	0.47	0.41	0.31	0.55
Group 3	0.98	0.88	1.10	0.97	0.86	1.09
Group 4	1.01	0.89	1.15	0.99	0.87	1.14
Group 5	0.74	0.60	0.91	0.79	0.64	0.97
Group 6	0.15	0.07	0.31	0.18	0.08	0.38

CI, confidence interval; HR, hazard ratio; MDA, Mediterranean diet adherence. [a] Adjusted for gender and age; [b] Adjusted for gender, age, smoke, and exercise.
